# Wound healing and inflammation genes revealed by array analysis of 'macrophageless' *PU.1 *null mice

**DOI:** 10.1186/gb-2004-6-1-r5

**Published:** 2004-12-23

**Authors:** Lisa Cooper, Claire Johnson, Frank Burslem, Paul Martin

**Affiliations:** 1 Department of Anatomy and Developmental Biology, University College London, London, WC1E 6BT, UK; 2 Pfizer Global Research and Development, Sandwich, Kent, CT13 9NJ, UK; 3 Departments of Physiology and Biochemistry, University of Bristol, Bristol, BS8 1TD, UK; 4 Current Address: Molecular Neuroscience Group, School of Medicine, University of Birmingham, Birmingham, B15 2TH, UK

## Abstract

**Background:**

Wound healing is a complex process requiring the collaborative efforts of different tissues and cell lineages, and involving the coordinated interplay of several phases of proliferation, migration, matrix synthesis and contraction. Tissue damage also triggers a robust influx of inflammatory leukocytes to the wound site that play key roles in clearing the wound of invading microbes but also release signals that may be detrimental to repair and lead to fibrosis.

**Results:**

To better define key cellular events pivotal for tissue repair yet independent of inflammation we have used a microarray approach to determine a portfolio of over 1,000 genes expressed across the repair response in a wild-type neonatal mouse versus its *PU.1 *null sib. The *PU.1 *null mouse is genetically incapable of raising the standard inflammatory response, because it lacks macrophages and functioning neutrophils, yet repairs skin wounds rapidly and with reduced fibrosis. Conversely, by subtraction, we have determined genes that are either expressed by leukocytes, or upregulated by fibroblasts, endothelial cells, muscle cells and others at the wound site, as a consequence of inflammation. To determine the spatial expression pattern for several genes in each cluster we have also performed *in situ *hybridization studies.

**Conclusions:**

Cluster analysis of genes expressed after wounding wild-type mice versus *PU.1 *null sibs distinguishes between tissue repair genes and genes associated with inflammation and its consequences. Our data reveal and classify several pools of genes, giving insight into their likely functions during repair and hinting at potential therapeutic targets.

## Abstract 2

To define the events in wound healing that are independent of inflammation, gene expression during wound healing was profiled in wild-type mice and *PU.1* null mice, which cannot raise the standard inflammatory response but which can repair skin wounds rapidly.

## Background

Much is known about the sequence of cell and tissue behavior that leads to repair of a mammalian skin wound [1,2] but we still have a rather incomplete knowledge of the portfolio of genes that drives these events. From late fetal stages onwards, tissue repair is always accompanied by a robust inflammatory response and this intimate association between wound healing and inflammation has made it difficult to dissect out the key elements of the repair process from those that are simply a consequence of inflammation and not necessary for healing. For this reason, and because adult skin healing is a complex process drawn out over several days to weeks, no systematic microarray analysis has yet been undertaken to encompass all those episodes from initial injury to the final sealing of the wound.

The compelling argument for performing such a study comes from microarray investigations of genes upregulated in fibroblasts in response to serum exposure. Cluster analysis of these results hints at the roles of hundreds of genes by the similarity of their temporal profile with genes whose function as part of the serum response cascade is well characterized [3,4]. To overcome the problems of extended wound repair time course and to distinguish repair genes from those involved in, or a consequence of, inflammation, we have developed an incisional wound model in neonatal mice where healing is rapid and largely complete by 24 hours and we have used this model to compare wound-expressed genes in wild-type mice versus *PU.1 *null sibs which are genetically incapable of raising an inflammatory response because they lack key leukocyte lineages.

*PU.1 *is an ETS family transcription factor that is crucial for several lineage decisions in hematopoetic cells; consequently, *PU.1 *null mice lack a number of hematopoetic cell types [5]. They are born with no macrophages or osteoclasts, and there is a late onset of neutrophil and T-cell development [5]. However, although there are no neutrophils or macrophages for recruitment to sites of tissue damage, neonatal *PU.1 *null mice can efficiently heal skin wounds [6]. Indeed, repair in the *PU.1 *null mice results in less indication of fibrosis and an altered cytokine and growth-factor profile compared to wild-type. For example, interleukin 6 (IL6) mRNA, which is robustly expressed at wild-type wound sites, is almost undetectable in *PU.1 *null wounds, and TGFβ1 mRNA, previously implicated in several fibrosis scenarios, is significantly reduced in *PU.1 *null wounds, as revealed by RNase protection analyses [6].

In this study we use Affymetrix GeneChip analysis of mRNAs collected at various time points after wounding of wild-type versus *PU.1 *null skin, to distinguish those key transcriptional events that are part of the tissue repair process but independent of whether or not there is an accompanying inflammatory response, from those genes that are 'inflammation dependent'. The latter are expressed either by inflammatory cells recruited to the wound, or upregulated by fibroblasts, endothelial cells, muscle cells and others at the wound site, as a consequence of inflammation.

Using cluster analysis we have grouped more than 1,000 wound-induced genes according to their temporal profiles, with each cluster having a unique temporal profile of expression that correlates with a clear physiological episode during the repair process. For a small sample of genes from each of these clusters, we show *in situ *hybridization data that also reveals spatial resolution.

## Results and discussion

For our wound model we chose neonatal mouse back skin which raises a robust inflammatory response to wounding that is not dissimilar to that seen at sites of tissue damage in adult skin, but which heals rapidly, such that incisional lesions are generally fully re-epithelialized by 24 hours. This compression of the repair process reduces the temporal 'noise' and thus the potential loss of gene-expression synchrony between wild-type and *PU.1 *null animals, which naturally will increase with time after the initial wound insult.

### Wounding of neonatal back skin results in rapid healing with or without an associated inflammatory response

**Figure 1:**
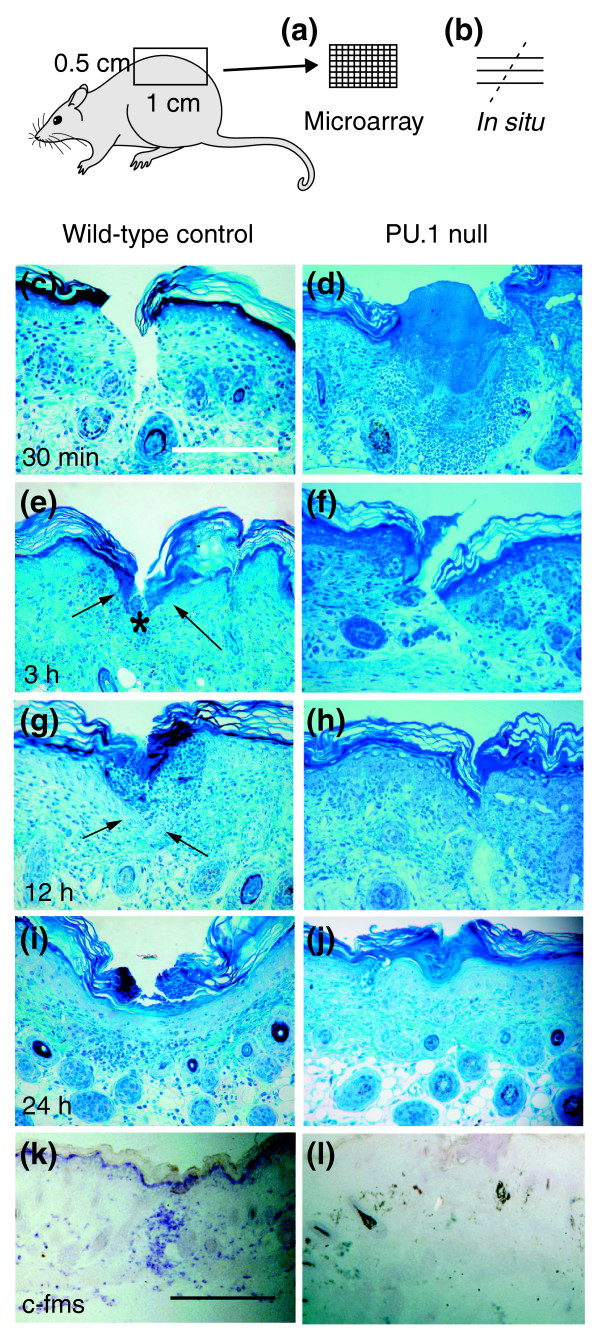
Wound histology. The location of skin wounds on the back of a neonatal mouse is shown. **(a) **For the array studies a series of criss-cross wounds were made so that all the skin cells were as close as possible to a wound edge for collection of wound RNA. **(b) **For *in situ *hybridization studies and immunohistochemistry we made a series of three incisional wounds, so that transverse sections (broken line) contained the profiles of several wounds. Resin histology through wild-type (left-hand column) and *PU.1 *null wounds (right-hand column) at **(c,d) **0.5 h, **(e,f) **3 h, **(g,h) **12 h and **(i,j) **24 h post-wounding. At all stages, arrows mark the epidermal wound edges, which are seen to have met and fused in both genotypes by 24 h. An asterisk (*) marks the migrating epithelial edge. **(k,l) ***In situ *hybridization using a macrophage-specific *C-fms *probe reveals large numbers of macrophages recruited to the granulation tissue in frozen sections through 24 h wounds in wild-type skin (k), while none are present in equivalent tissues of the *PU.1 *null mouse (l). Scale bars = (c-j) 400 μM; (k,l) 250 μM.

Resin histology of healing incisional wounds in neonatal mouse skin reveals closure of the wound commencing within 3 hours of the lesion; by 24 hours, the epidermal wound edges have generally met and fused along much, if not all, of the length of the wound. This is true for both wild-type neonates and for *PU.1 *null sibs, with the only obvious differences apparent in the histology being an absence of inflammatory cells in the *PU.1 *null wounds (Figure 1c-j). As previously described, *in situ *hybridization studies using a *c-fms *macrophage-specific probe reveal large numbers of these cells drawn to the wound connective tissue just beneath the epidermal fusion seam at 24 hours after wounding wild-type skin, but their complete absence in *PU.1 *null equivalent wound sections (Figure 1k,l). The same difference, although with an earlier temporal profile, is observed if wounds of wild-type and *PU.1 *null skin are probed with histochemical stains that reveal neutrophil influx (data not shown). These wound dynamics lead us to believe that the neonatal mouse skin wound model provides a good opportunity to analyze the transcriptional events that regulate the various tissue-repair episodes from initial activation steps through to the 'stopping' signals that occur when the tissue defect has been filled in, both in the presence and absence of an inflammatory response.

### More than 1,000 genes are differentially expressed post-wounding

For microarray comparison, a consistent series of horizontal and vertical incisional (criss-cross) wounds were made to the back skin of 2-day-old neonatal *PU.1 *null mice and their wild-type littermates. Each time-matched pair (*PU.1 *null and wild-type) were chosen from the same litter to reduce the possibility of differential expression 'noise' due to environmental differences. Wound tissues were harvested at either 30 minutes to identify immediate early genes, 3 hours for early tissue repair effector genes, and 12 or 24 hours to reveal later tissue repair effectors as well as inflammatory genes. Total RNA was then extracted and hybridized to Affymetrix GeneChips, and differentially expressed genes were identified by comparison of expression levels for each time point with unwounded skin samples that served as baseline controls. Genes were selected if transcript levels exceeded a twofold increase over either the unwounded baseline, or between time points, or between the wild-type and *PU.1 *null wounds. On the basis of these criteria, 1,001 genes were identified as wound-induced (see Additional data file 1 for an annotated database of all these genes together with full details of expression levels at all time points).

### Cluster analysis to group these genes reveals temporal profiles that correlate with distinct physiological episodes in the repair process

Cluster analysis with Spotfire Array Explorer 3.0 software was used to organize the 1,001 wound-induced genes into groups according to the cosine coefficient similarity measurement; this includes within a group all those genes that have a similarly shaped temporal profiles, regardless of the levels of gene expression. Nine clusters were identified in this way, and of these, seven correlated with clear episodes in the repair process. The other two had profiles that, as far as we can tell, do not correspond to any currently understood step in the repair process and so were discarded for further analysis, although they appear in our supplementary data (see Additional data file 2 for median graphs of these clusters). Of the seven clusters associated with known repair episodes, five contain one or more known genes with good functional associations to that repair episode, and this encourages us to name each cluster according to that physiological episode. This does not provide definitive proof of function for any gene in that cluster, but it gives the best opportunity to predict function, particularly for expressed sequence tags (ESTs) with no further sequence information.

### Four clusters have profiles that are independent of an inflammatory response

**Figure 2:**
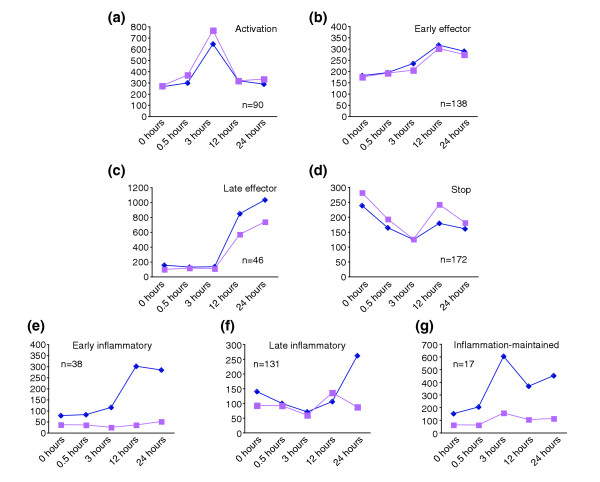
Median temporal profile graphs of identified repair and inflammation clusters. Line graphs displaying the median level of absolute mRNA expression (*y*-axis) at each time point: 0, 0.5, 3, 12 and 24 h (*x*-axis), for genes within each of the four repair clusters and the three inflammation clusters, giving representative temporal profiles for the cluster. Pink lines represent the temporal profiles of expression for the *PU.1 *null wound site, blue lines those for the wild-type wound site. **(a-d) **The inflammation-independent gene clusters: (a) activation; (b) early effector; (c) late effector; (d) stop. **(e-g) **The inflammation-dependent clusters: (e) early inflammatory; (f) late inflammatory; (g) inflammation-maintained. The scale of absolute expression levels along the *y*-axis varies according to the maximum levels of expression in each cluster.

Four clusters of genes have profiles that are largely independent of inflammation. Genes in these clusters are expressed with similar profile whether wounds are in wild-type skin, where there is an influx of inflammatory cells, or in *PU.1 *null skin, where there is none. In both these situations there is full and complete repair, and so we propose that these four clusters represent the basic repertoire of repair genes that are activated during the repair response. Figure 2 shows line graphs that display the temporal profile of the median expression levels at each time point to give a representation of that cluster and these have been termed the 'activation' (Figure 2a), 'early effector' (Figure 2b), 'late effector' (Figure 2c) and 'stop' (Figure 2d) clusters. The number of genes found in each cluster is displayed on each graph.

### Three gene clusters correlate with various phases of the inflammatory response

Three further clusters of genes represent expression profiles that correlate with the onset of inflammation and thus we consider them inflammation-associated genes. In neonatal animals, the inflammatory response is generally induced by 12 hours and is well established by 24 hours post-wounding. Two of these inflammation-associated gene clusters contain genes that are not expressed in unwounded skin or at early stages of repair; rather, they are upregulated in wild-type skin directly coincident with the onset of the wound inflammatory response but are generally not upregulated in the *PU.1 *null wound site at any stage. We have called these two groups of genes, the 'early inflammatory' cluster (Figure 2e) and the 'late inflammatory' cluster (Figure 2f). A third cluster does not display the standard inflammatory response profile as typified by the early and late inflammatory clusters. Rather, this cluster contains genes that are expressed at early stages of repair in both *PU.1 *null and wild-type mice but, whereas expression appears to increase in the wild-type wound coincident with the inflammatory response, the same genes are downregulated in the *PU.1 *null wound, where there is no inflammatory response; we have called this group of genes the 'inflammation-maintained' cluster (Figure 2g).

### Nearly 100 genes are expressed with an immediate early gene profile at the wound site

**Figure 3:**
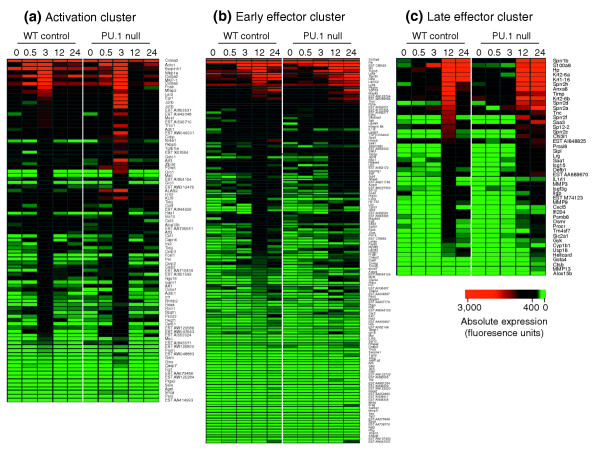
Heatmaps for activation and effector clusters. Color depiction of the temporal profiles of mRNA intensity during the 24 h repair period for genes in **(a) **the activation cluster, **(b) **the early effector cluster and **(c) **the late effector cluster. Higher levels of expression are indicated by progressively brighter shades of red, and lower expression levels by increasingly brighter shades of green. The scale bar indicates absolute expression as a measure of fluorescence units. Genes are ordered with the most highly expressed first. Gene names are shown to the right of the maps and further bioinformatics data for each can be found in Additional data file 1. Expression levels for the *PU.1 *null wound site at 0, 0.5, 3, 12 and 24 h are shown on the right and the equivalent expression levels for the wild-type (WT) wounds on the left.

One of the most clear-cut clusters of genes is of those whose temporal expression profiles are suggestive of a transient, immediate early response to wounding. These genes show almost identical profiles, whether in the wild-type or *PU.1 *null situation, and thus are independent of an inflammatory response. We have named this group the activation cluster, as many will be kick-start activators of the various cell behaviors that together comprise the wound-healing process. This cluster is dominated by transcription factors and contains several well known immediate early genes, such as *Egr1 *(*Krox 24*), *JunB*, *Myc*, and *I-Kappa-Bα *(*Nfkbia*). We present a heatmap for the 90 genes in this cluster arranged with the most highly expressed at the top of the map (Figure 3a). Heatmaps provide a visual representation of temporal profiles only, and so for a small sample of these genes we also include *in situ *hybridization data on wounded skin sections to illustrate which cells and tissues express that particular gene. This spatial expression profile reveals expression in the *in vivo *setting, giving clues to the function of that gene during repair.

**Figure 4:**
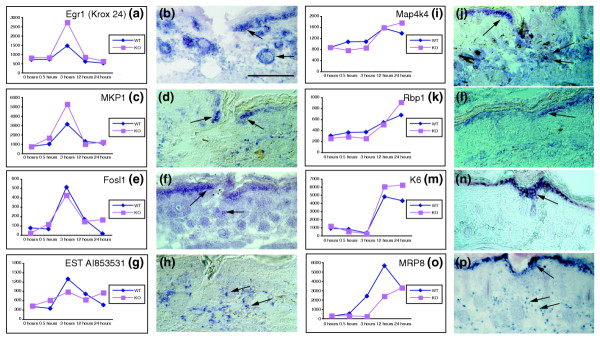
Temporal and spatial expression profiles of sample genes from the activation and effector clusters. Temporal and spatial profiles of the **(a-h) **activation and **(i-p) **effector clusters. The line graphs display temporal expression: absolute expression levels (*y*-axis) at each time point (*x*-axis) with both *PU.1 *null (pink) and wild-type profiles (blue). The *y-*axis range varies depending on the expression levels for each gene. The photomicrographs show *in situ *hybridization on 3 h (b,d,f,h) and 12 or 24 h (j,l,n,p) frozen wild-type wounds. (a,c,e,g) Temporal profiles of each of the activation genes show a rapidly induced but transient expression peak at 3 h in both *PU.1 *null and wild-type wounds. (b) *Krox24 *is expressed by wound margin epidermal cells extending back 10-12 cell diameters from the wound edge and also by associated hair follicles (arrows). (d) *MKP-1 *is expressed by the first 5-8 front-row keratinocytes and a subset of dermal fibroblasts (arrows). (f) High levels of *Fosl1 *expression in wound margin epidermal cells and weaker expression in damaged hair follicles (arrows). (h) EST GenBank accession number AI853531 appears to be expressed by wound fibroblasts (arrows). (i,k,m,o) The early and late effector gene samples all exhibit expression profiles with upregulation either at 12 h (i,k), or 24 h (m,o), whether in *PU.1 *null or wild-type wound tissues. (j) *Map4k4 *is expressed up to 10-12 cell diameters from the wound edge and in dermal fibroblasts (arrows). (l) *Rbp1 *is expressed in epidermal cells approximately 15 cell diameters from the wound site. (n) *K6 *expression is restricted to 10-12 rows of wound edge keratinocytes. (p) *MRP8 *has a rather similar keratinocyte expression to *K6*, but is also expressed to a lesser extent in leukocytes in wild-type wounds (arrows). Scale bar = 400 μm.

*Krox24 *(Figure 4a) has previously been shown to be transiently induced in both embryonic and adult mouse wounds [7]. *In situ *hybridization reveals *Krox24 *to be expressed by those epidermal cells extending back 10-12 cell diameters from the cut edge of neonatal wounds and all the associated hair follicles within this zone also (Figure 4b).

MKP-1 (Figure 4c) is a dual-specificity phosphatase with close homology to *Drosophila *puckered, which has been shown genetically to be key in braking the Jun N-terminal kinase (JNK) cascade activated during morphogenetic episodes such as dorsal closure in the fly embryo [8]. *In situ *hybridization shows that the front few rows of wound epidermis express *MKP-1*, although expression extends less far back from the wound edge than for *Krox24 *(Figure 4d). By analogy to *Drosophila *morphogenetic episodes, it may be that MKP-1 operates as suppressor of MAP kinase (MAPK) signaling by phosphorylation of extracellular-regulated kinases 1 and 2 (ERK1 and 2), and so may actually function as a brake on the earliest tissue movements activated at the wound site.

Expression of Fos-like antigen 1 (*Fosl1*, *Fra1*), has previously been associated with epithelial migrations during tumorigenesis but has not been analyzed in a wound-repair model [9,10]. *Fosl1 *has a classic activator temporal profile (Figure 4e) and a similar spatial profile to *Krox24*, with high levels of expression in wound-margin epidermal cells but somewhat weaker expression in damaged hair follicles at the wound site (Figure 4f). Its close relative *c-fos *has previously been shown to be upregulated during repair of embryonic skin wounds [11], and *in vitro *studies show that blocking wound-induced *fos *induction may hinder cell migration [12].

Because cluster analysis allows us to group genes together that are likely to have similar functions [13], the temporal profiles of, as yet, uncharacterized ESTs in the activation cluster implicates them as having an immediate-early activator function during repair. A good example of such a gene is EST GenBank accession number AI853531 (Figure 4g), which is weakly homologous to human *Mitogen-Inducible-Gene-6 *(*Mig-6*, *Gene 33*). The exact function of Mig-6 remains elusive but it has been shown to interact with Cdc42, a member of the Rho family of GTPases, via the activation of stress-activated protein kinases (SAPKs) [14]. *In situ *hybridization reveals clear expression of this gene in wound fibroblasts (Figure 4h); together with its potential Cdc42 effector status and its induction in quiescent fibroblasts upon mitogenic stimulation and expression in many human cancer cell lines [15], this suggests that Mig-6 may mediate a fibroblast migration signal. The remaining genes in the activation cluster all have very similar temporal profiles, suggesting that they too may have important roles in activating or modulating early cell behavior at the wound edge.

### A further 200 genes are also expressed independently of inflammation, but with later onset and a less transient time course

Two further clusters of genes have increased expression levels post-wounding in a manner that is also inflammation-independent but where expression occurs at a later time than with the activation genes. The profiles of these two clusters are temporally distinct from one another and so we have called them the early effector and late effector clusters. Between them they contain 184 genes that fit the expected profile of genes that might direct re-epithelialization and granulation tissue assembly events. The temporal profiles of all these genes can be seen by heatmap in Figure 3b (early effector cluster) and Figure 3c (late effector cluster). These two clusters contain varied types of tissue repair effectors such as tissue remodelers, genes encoding extracellular matrix (ECM) proteins, those involved in the signaling machinery and structural genes required for cell migration. Again, we provide here several examples of genes within these clusters with accompanying *in situ *hybridization data to provide an insight into the spatial localization of some genes in these clusters.

Map4k4, a member of the serine/threonine protein kinase family that activates the JNK and MAPK signaling pathways in response to stress signals, cytokines and growth factors [16], is a member of the early effector cluster. The temporal profile (Figure 4i) and expression of *Map4k4*, in both keratinocytes up to 10-12 cell diameters from the wound edge and a subset of dermal fibroblasts extending a similar distance back from the wound edge (Figure 4j), confirms the activation of this intracellular signaling cascade at sites of tissue repair. The JNK pathway has recently been shown to have a role in Paxillin regulation during fibroblast migrations triggered by *in vitro *scratch wounds [17], and so expression of *Map4k4 *is also suggestive of a cell migratory regulatory role for this signaling pathway in keratinocytes and fibroblasts during *in vivo *repair.

Also in the early effector cluster, retinol binding protein-1 (Rbp1), a Fabp/p2/Crbp/Crabp family retinol transporter is expressed in wound epidermal cells approximately 15 cell diameters back from the wound site (Figure 4k and 4l). This suggests a role for retinoids in re-epithelialization of the wound, and indeed, there is some evidence that these molecules can trigger epidermal proliferation via heparin-binding epidermal growth factor (HB-EGF) expression in suprabasal epidermal cells [18].

Typifying the late effector profile is *Keratin 6 *(*K6*), a classic wound-induced gene [19] (Figure 4m). *K6 *encodes a nonconventional keratin which is thought to facilitate the packaging up of other intermediate filaments in activated keratinocytes, so that these cells can migrate forward to re-epithelialize the wound [19]. High levels of expression of *K6 *by the front 10-12 rows of wound-edge keratinocytes were confirmed by *in situ *hybridization (Figure 4n).

Interestingly, another member of the late effector cluster, the intracellular Ca^2+^-binding protein MRP8 (S100A8) is expressed in a similar temporal and spatial pattern to *K6 *(Figure 4o and 4p). MRP8 binds to keratin filaments as an MRP8/14 heterodimer in a Ca^2+^dependent manner [20,21] and is postulated to interact with these keratin filaments and guide cytoskeletal rearrangements during tissue repair [22]. The temporal and spatial coexpression of *K6 *with *MRP8 *may highlight a relationship between them and as such reveals another advantage of cluster analysis - the ability to identify potential interactions between genes and genetic pathways within the same cluster.

Not all functionally related genes cluster together, however. The heterophilic binding partner of MRP8 is MRP14, which does not appear in the same cluster but rather is expressed within the early inflammation cluster (see later), since, in addition to keratinocyte expression, it is expressed at high levels by wound leukocytes. As both the MRP8/MRP14 heterodimer and a homodimer, MRP8 is a potent chemoattractant [22,23] and, interestingly, the MRP8/14 heterodimer also has an entirely different role, operating as a wound antimicrobial factor, although the MRP14 subunit seems to be responsible for this activity [24]. The pleiotropic activities of MRP8/MRP14 may reflect different functions of monomeric versus complexed subunits.

### A final cluster of inflammation-independent genes may indicate players in the 'contact inhibition' stopping process

At the end of the repair process many of the cell behaviors that drive repair - such as migration and proliferation - clearly need to cease as tissues re-establish approximately their pre-wound state. This will be a gradual process and yet we might expect to see such genes depressed during the repair period and becoming upregulated as wound edges meet and closure is finishing. We see a cluster of genes with exactly this profile, suggesting that some of these genes are re-expressed to control the later stages of repair. We have loosely termed this the stop cluster. Because of their known biology, several genes in this cluster make ideal candidates for players in the processes of contact inhibition and epithelial fusion that occurs as cells from the two epidermal wound fronts confront one another.

**Figure 5:**
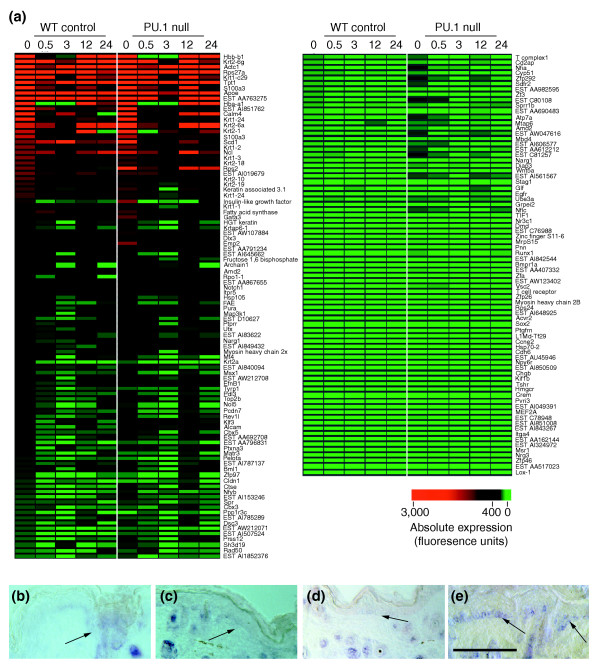
Heatmap and *in situ *hybridization data for genes in the stop cluster. **(a) **The temporal expression profiles of genes of the cluster are represented by a heatmap. The highest levels of expression are indicated by the brightest shades of red, while lower expression levels are represented by progressively brighter shades of green, as indicated by the scale bar. Genes are ordered with the most highly expressed first, and gene names are shown to the right of the maps. **(b-e) **A temporal series of *in situ *studies revealing expression of one gene in this class, *Notch*, at 0.5 h (b), 3 h (c), 12 h (d) and 24 h (e), showing how mRNA levels in the leading-edge keratinocytes appear to dip during the period of re-epithelialization and then increase again coincident with the time at which epidermal fronts contact one another. Arrows highlight region of gene expression. Scale bar = 100 μm.

The Eph receptors and their ligands, the ephrins, have features that might make them ideal for sensing and responding to stop cues. *In vitro *studies show that both ligand- and receptor-bearing cells become activated upon cell-cell contact [25,26], and this interaction leads to a repulsive response by receptor-expressing growth cones during the developmental wiring of the nervous system [27]. Further evidence for ephrin-mediated control of epithelial sheet movement and fusion comes from studies in *Caenorhabditis elegans*, where Eph receptor mutants display defects in the movement of epidermal cells over neuroblasts, and in Eph knockout mice, where various morphogenetic epithelial fusions fail, leading, for example, to cleft palate and hypospadius [28,29]. All these results suggest that the transcriptional regulation of EphB1 revealed in the heatmaps for our stop cluster (Figure 5a) may reflect a functional role in the stopping or final fusion episodes of wound re-epithelialization.

Similarly, the expression levels of the receptor Notch also dip and rise during the repair period, and *in situ *hybridization studies reveal that this transcriptional regulation is also occurring within leading wound-edge epidermal cells (Figure 5b-e). Notch has exceptionally complex biology with several ligands, including Delta and Serrate, and is a widely used as a signaling cassette at various stages of embryogenesis, and has been shown to be downregulated in several invasive tumors [30]. In *Drosophila*, Notch signaling has been implicated in the contact inhibition and fusion events that occur during dorsal closure at the end of embryogenesis (A. Martinez-Arias, personal communication), and during gut cell migratory episodes, which are also dependent on transcriptional activation of the *short stop *gene [31], the mammalian orthologue of which, Actin crosslinking family 7 (ACF7), is another member of our wound stop cluster.

Several other genes within the stop cluster have characteristics that indicate they may be involved in sensing contact-inhibition cues or be downstream of these signals and operate to adhere epidermal fronts together. They include genes for Plexin 3 (Plxn3), a member of the plexin family of semaphorin receptors [32], Desmocollin 3 (Dsc3), which is a cadherin component of intercellular desmosomal junctions [33] and ACF7, a cytoskeletal linker protein [34].

As with the other clusters, suggestive biology is no proof of function, and it is worth noting that several other genes with this temporal profile do not have biology suggestive of a role in these late stages of wound healing. We feel that this cluster, more than any other, can only hint at function, and definitive function testing using knockout or knockdown assays will be necessary to investigate any speculative roles in the repair process.

### Expression of 200 genes at the wound site is dependent on the inflammatory response

**Figure 6:**
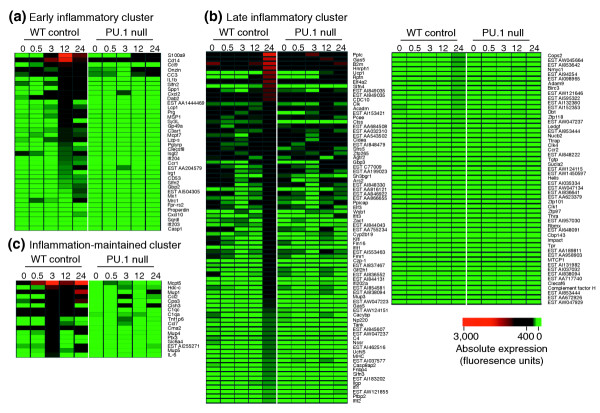
Heatmaps for inflammation-dependent genes. **(a-c) **Heatmaps of the temporal profiles of mRNA intensity during the 24 h repair period for inflammation-dependent genes in wild-type and *PU.1 *null wounds. (a) The early inflammatory cluster corresponds to the earliest onset of the inflammatory response with a temporally later induction seen in the late inflammatory cluster (b). (c) The inflammation-maintained cluster also appears to be regulated by the inflammatory response. Highest levels of expression are indicated by progressively brighter shades of red and lower expression levels represented by progressively brighter shades of green, as shown by the scale bar. Genes are ordered, for each cluster, with the most highly expressed first, and gene names are shown to the right of the maps.

A comparison of those genes expressed during the repair process in wild-type versus *PU.1 *null mice reveals most clearly genes that are dependent on the presence of an inflammatory response at the wound site. The heatmaps for early and late inflammatory gene clusters strikingly reveal robust expression in wild-type wounds, but little or no expression in the *PU.1 *null mice for these genes (Figure 6). Together, the early and late inflammatory clusters comprise 169 genes that are not expressed in unwounded wild-type skin or at early stages of repair but appear to be upregulated in the wild-type wound directly, coincident with the onset of the inflammatory response. The early inflammatory cluster typically contains genes whose expression is upregulated rapidly in the wild-type, often reaching a peak by 12 hours (Figure 6a), coincident with the influx of neutrophils to the wound site. In the late inflammatory cluster, expression typically peaks a little later, at 24 hours post-wounding (Figure 6b), more suggestive of a link to the later influx of macrophages. A further 17 genes are initially expressed at both the wild-type and *PU.1 *wound site, but are maintained at high level only in the wild-type wound, where there is an influx of leukocytes. In *PU.1 *null wounds, where there is no such influx, these genes are only transiently expressed. We assume that expression of these inflammation-maintained genes (Figure 6c) is directly or indirectly dependent on signals released by inflammatory cells.

### Inflammation-dependent genes may be expressed by leukocytes or by host cells as a 'response signature' to inflammatory signals

Genes that are expressed only in wild-type wounds and whose temporal expression patterns are coincident with the influx of neutrophils and/or macrophages will include those genes that are constitutively expressed by one or both of these lineages, or genes that are upregulated as part of the leukocyte activation state, or may be expressed by cells other than the invading leukocytes as a downstream consequence of host fibroblast, endothelial and muscle cells being exposed to signals from these leukocytes. We present a selection of *in situ *hybridization studies to illustrate each of these scenarios as revealed by very distinct classes of spatial expression pattern.

#### Early inflammatory cluster

**Figure 7:**
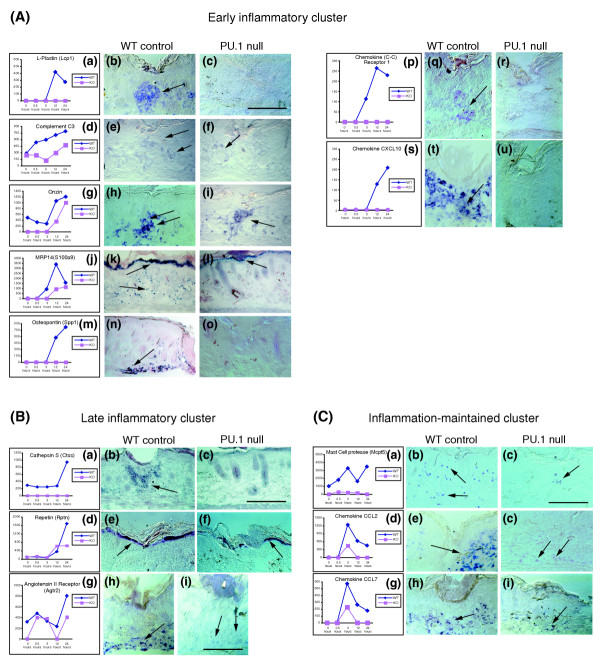
Temporal and spatial expression profiles of sample genes from the three inflammation-dependent clusters. Temporal and spatial profiles of the **(A) **early inflammatory, **(B) **late inflammatory and **(C) **inflammation-maintained clusters. Line graphs display absolute temporal expression levels (*y*-axis) at each time point (*x*-axis) for both *PU.1 *null (pink) and wild-type (blue) wounds. *y*-axis expression levels vary according to individual gene expression levels. *In situ *hybridization studies of (A) 12 h, (B) 24 h or (C) 3 h frozen sections illustrate the contrasting expression patterns of each of these classes of genes in wild-type (WT) versus *PU.1 *null wounds. **(Aa,d,g,j,m,p,s) **In the early inflammatory cluster, expression in wild-type wounds peaks at 12 h but is absent or significantly reduced in *PU.1 *null wounds. **(Ab,c) ***In situ *studies show *L-plastin *to be expressed by activated leukocytes in the wild-type only (arrow). **(Ae,f) **Faint expression of *C3 *is seen in both genotypes (see arrows). **(Ah,i) ***Onzin *expression appears to be in the same cells within the connective tissue in both genotypes. **(Ak,l) **Both keratinocytes and leukocytes (arrows) express *MRP14 *in the wild-type but only keratinocyte expression (arrow) is seen in the *PU.1 *null wound. **(An,o) ***Osteopontin *displays a possible 'fibrosis' gene spatial profile with expression in deep dermal cell layers (arrow) in the wild-type only. **(Aq,r) ***CCr1 *is expressed only in the wild-type wound, in cells whose clustered location suggests they are one of the leukocyte lineages.**(At,u) **Expression of *CXC10 *is broad and throughout the wound connective tissue of wild-type wounds (arrow) suggesting that expressing cells are wound fibroblasts. (B) In the late inflammatory cluster, expression in wild-type wounds appears to peak beyond 12 h in wild-type wounds and is absent or reduced in *PU.1 *null wounds. **(Bb,c) **Expression of *Cathepsin S *is seen in activated leukocytes in the wild-type only (arrow). **(Bd) ***Repetin *is expressed by both genotypes but to a lower level in the *PU.1 *null. **(Be,f) ***Repetin *is only upregulated by keratinocytes but is not restricted only to wild-type wounds (arrows). **(Bh,i) **Expression of the potential fibrosis gene *Angiotensin II Receptor 1 *is seen in deep dermal cell layers of wild-type and, to a significantly reduced level, *PU.1 *null wounds (arrows). (C) In the inflammation maintained cluster, the expression profiles suggest that while these genes may be initially expressed in *PU.1 *null wounds, persistent expression requires the presence of an inflammatory response as in the wild-type wound situation. **(Cb,c) **Expression of *Mcpt5 *is seen at both wound sites (arrows) in scattered cells throughout the wound connective tissue. **(Ce,f) ***CCL2 *appears to be expressed by host wound cells at both wound sites (see arrows). **(Ch,i) ***CCL7 *is expressed in an almost identical temporal and spatial profile to *CCL2*. Scale bars = 400 μm (Aa-o, Ba-f, C) and 250 μm (Ap-u and Bh,i).

L-plastin (Lcp1) is a pan-leukocyte, calcium-dependent, actin-bundling protein that has previously been implicated in macrophage activation and migration, although it is also overexpressed in many types of malignant human tumors [35]. It is first expressed in the wild-type wound coincident with the early stages of the wound inflammatory response, with a peak of expression at 12 hours post-wounding; our temporal data indicate no expression at any stage in *PU.1 *null wounds (Figure 7Aa). *In situ *hybridization studies reveal intense expression by leukocytes clustered within the wild-type wound site but no expression in surrounding skin (Figure 7Ab), and they confirm the absence of expression in *PU.1 *null wounds (Figure 7Ac). The wound-restricted expression pattern of *L-plastin *suggests that expression of this gene is limited to activated leukocytes only.

Also expressed by leukocytes in the early inflammatory cluster are C3, a key component of the classical and alternative complement pathways, and its receptor, C3R. *C3 *is expressed at similar levels in unwounded *PU.1 *null and wild-type skin, but whereas expression is rapidly upregulated by 30 minutes post-wounding and continues until 24 hours in wild-type wounds, upregulation of *C3 *is delayed and much weaker in the *PU.1 *null wound (Figure 7Ad-f). This delay in *C3 *expression suggests that inflammation has a significant role in raising and maintaining a rapid complement response at the wound site.

*Onzin *also appears as a member of the early inflammatory cluster; it encodes a leukaemia-inhibitory factor-regulated protein that has previously been identified in a screen for genes controlling inflammatory dermatitis [36]. Unwounded wild-type skin expresses *Onzin *at low levels but it is completely absent in *PU.1 *null, unwounded skin and remains so until 12 hours post-wounding, when it is upregulated, but to a much lesser extent than in wild-type (Figure 7Ag). *In situ *hybridization studies reveal a rather similar expression pattern in both wild-type and *PU.1 *null wounds (Figure 7Ah,i). This suggests that *Onzin *might be expressed in wild-type skin by resident inflammatory cells and in the *PU.1 *null wound, either by inflammatory cells whose development is delayed, such as T cells, or that there may be an alternative or compensatory mechanism of gene regulation in non-inflammatory cells at the wound site.

As discussed previously, both the genes for MRP8 and its binding partner MRP14 are upregulated by wound-edge keratinocytes. Both are also expressed by leukocytes, and in the case of *MRP14 *this expression predominates and leads to cluster separation of the two genes, with *MRP14 *categorized as part of the early inflammatory cluster. In the wild-type wound, it is expressed from 3 hours, with expression peaking at 12 hours post-wounding, whereas in the *PU.1 *null, expression does not begin until 12 hours post-wounding and levels are much reduced compared with wild-type (Figure 7Aj). *In situ *hybridization clearly shows *MRP14 *to be expressed, in addition to expression in keratinocytes, in the region of the wound populated by inflammatory cells in the wild-type only (Figure 7Ak,l), and indeed, previous experiments suggest that both neutrophils and macrophages express *MRP8 *and *MRP14 *[22].

It may be that genes expressed by host connective-tissue cells at the wound site as a consequence of inflammatory signals are detrimental to healing and lead to some of the imperfect aspects of repair seen in adult healing such as fibrosis and scarring. One candidate for such a gene is *Osteopontin *(*Spp1*, *minopontin*), encoding a glycoprotein that can mediate cell-matrix interactions via the engagement of a number of adhesive receptors (reviewed in [37]). Previous wound-healing studies on *Spp1 *null mice report differences from wild-type in that repair is characterized by abnormal macrophage debridement and abnormal maturation of collagen bundles [38]. *Osteopontin *has a clear inflammation-related profile (Figure 7Am) and *in situ *hybridization reveals an unusual pattern of expression at the wild-type wound site, with some expression by a subset of leukocytes but with most positive cells located in what appears to be the deep dermal or muscle layers of the wound region (Figure 7An,o).

Both the early and late inflammatory clusters contain chemokine and growth factor receptors unique to leukocytes, and presumably used by these cells to detect various chemotactic cues that will guide them to the wound site. For example, the gene for chemokine receptor 1 (*CCr1*), a receptor for several chemokines including MIP-1α, CCL5 and Scya7, is expressed as early as 3 hours post-wounding, with expression levels peaking by 12 hours. There is no expression at the *PU.1 *null wound site (Figure 7Ap). *In situ *studies show *CCr1 *to be expressed in the wild-type wound by leukocytes recruited to the wound site (Figure 7Aq,r). As well as chemokine receptors, chemokines themselves are found in these clusters. *CXCL10 *(*IP-10*) encodes an α-chemokine that functions as a potent chemoattractant for macrophages and T cells, and is upregulated by 12 hours in wild-type wounds but is absent in *PU.1 *null wounds (Figure 7As). *In situ *studies reveal intense staining by what could be either leukocytes or host fibroblasts at the wild-type wound site (Figure 7At,u). Either this chemokine is an amplifying chemotactic signal expressed by leukocytes to draw in further leukocytes, or its expression is triggered in fibroblasts, but only if they receive signals from the first influx of neutrophils.

#### Late inflammatory cluster

*Cathepsin S *is a typical gene of the late inflammatory cluster, being highly upregulated at 24 hours post-wounding in the wild-type, but with no expression in the *PU.1 *null wound (Figure 7Ba). Cathepsin S is one of a large family of leukocytic proteases - this one largely macrophage-specific - that catalyze the remodelling of ECM proteins. *In situ *hybridization studies in the wild-type wound show *Cathepsin S *to be expressed by macrophages clustered around the wound site, but also by cells in the dermis at skin sites well away from the wound (data not shown), suggesting that it is constitutively expressed by cells of the monocyte lineage, rather than being part of the macrophage activation profile. No expression of *Cathepsin S *is seen in wounded or unwounded skin of the macrophageless *PU.1 *null mouse (Figure 7Bb,c).

*Repetin *is an epidermal differentiation gene and a member of the fused gene subgroup of the S100 family that encodes multifunctional epidermal matrix proteins [39]. This temporal profile at the wound site implicates *Repetin *as being responsive to inflammatory signals (Figure 7Bd), and yet *in situ *hybridization studies reveal it is not expressed by inflammatory cells, but rather by leading-edge keratinocytes in both wild-type and *PU.1 *null wounds (Figure 7Be,f). While not absolutely dependent on inflammatory signals, it appears that *Repetin *expression by wound keratinocytes is significantly enhanced by inflammatory cues. As several studies have shown somewhat enhanced rates of re-epithelialization where one or more components of the inflammatory response are reduced during healing [6,40,41], it is tempting to speculate that genes like *Repetin*, which are upregulated in the wound epidermis in response to inflammatory signals, may in some way retard the re-epithelialization process.

As with the early inflammatory cluster, there are several genes in the late inflammatory cluster that may directly or indirectly, via their effects on signaling pathways, be responsible for wound fibrosis. The angiotensin II receptor has previously been implicated in mediating the fibrotic response in several tissue injury situations, such as myocardial infarction [42-45]; its gene is also a member of the late inflammatory cluster but is expressed at both wild-type and *PU.1 *null wounds. Expression is clear in both wild-type and *PU.1 *null wounds but significantly higher in the wild-type (Figure 7Bg). The spatial expression pattern of *Angiotensin II receptor *is reminiscent of *Osteopontin *in the early inflammatory cluster, with the brightest staining in the deep dermal or muscle layer of the wild-type wound and only very faint expression seen at the *PU.1 *null wound site (Figure 7Bh,i). Presumably, a subset of genes found in these inflammatory clusters, which are upregulated by host granulation tissue lineages rather than by leukocytes, may turn out to be markers, or direct regulators, of the fibrotic response that is routinely activated in adult wound granulation tissue. Clearly, therapeutic reduction of the products of these genes at the wound site might result in the reduction of wound fibrosis.

#### Inflammation-maintained cluster

A final cluster of genes appears to be regulated by the inflammatory response in that they are generally expressed at early stages post-repair in both *PU.1 *null and wild-type mice, but, whereas their expression subsequently diminishes in the *PU.1 *null mouse, expression is maintained, or increases, coincident with the inflammatory response in wild-type wounds. This temporal expression profile is most clearly visualized from heatmap data (Figure 6c). Some of the genes in this cluster implicate mast cells in the recruitment of other leukocyte lineages which then amplify the inflammatory signal. For example, Mast Cell Protease 5 (Mcpt5) is a serine chymase stored in the secretory granules of mast cells and acts as a potent chemoattractant [46]. *Mcpt5 *is rapidly and transiently upregulated immediately post-wounding and by 12 hours is back to near basal levels in the wild-type wound. However, it is secondarily upregulated at 24 hours. Expression is also clear at the *PU.1 *null wound site as an immediate response but levels remain low and there is no second peak of expression (Figure 7Ca). *In situ *hybridization studies show expression by scattered cells within the wild-type wound, with low levels of expression detected at the *PU.1 *null wound site also (Figure 7Cb,c). These data suggest that *Mcpt5 *is initially expressed independently of signals from macrophages and neutrophils, but that leukocytes are subsequently responsible for a secondary expression, either directly by expressing *Mcp5 *themselves, or indirectly by triggering expression in another cell type, possibly supplying cues that reinforce expression by mast cells or prevent their dispersal from the wound site.

Chemokines are also represented in this inflammation-maintained cluster. CCL2 and CCL7 are C-C chemokines with roles in directing the cellular composition of the inflammatory response. They are upregulated at 3 hours with expression tailing off by 24 hours post-wounding in the wild-type. In the *PU.1 *null wound, *CCL2 *and *CCL7 *are also upregulated at 3 hours but to a lesser degree than in the wild-type, and unlike in the wild-type, expression is immediately downregulated, so that by 12 hours post-wounding there is a complete absence of expression (Figure 7Cd,g). This suggests that expression is enhanced and maintained in the wild-type by the presence of macrophages and neutrophils, whereas in the *PU.1 *null wound, initial expression is independent of these leukocytes but without them expression cannot be amplified and maintained. Our *in situ *studies suggest that these chemokines are expressed by host wound connective-tissue cells rather than leukocytes at both the wild-type and *PU.1 *null wound sites (Figure 7Ce,f, and Ch,i).

## Conclusions

Here we report an Affymetrix GeneChip microarray study of *in vivo *wound healing using a neonatal mouse wound model where all phases of the repair process are compressed into a 24-hour period. Cluster analysis of wild-type wounds versus those of *PU.1 *null mice that are genetically incapable of raising an inflammatory response allow us to distinguish repair genes from those involved in, and a consequence of, wound inflammation.

Several previous studies have modeled wound healing *in vitro *by exposure of fibroblasts to serum, as tissue damage to blood vessels *in vivo *leads to exposure of connective-tissue cells to blood serum [3]. Our results show that for the earliest phases of the repair process, this model does indeed mirror the *in vivo *repair response. When we consider only those genes present on both experimental microarrays, we see that more than 40% of the genes that are upregulated with an immediate early profile at the wound site have previously been shown to have similar temporal profiles after *in vitro *activation of fibroblasts. Other *in vitro *models of repair also turn out to be rather good predictors of the *in vivo *response. For example, exposure of keratinocytes to keratinocyte growth factor (KGF) *in vitro *reveals many of the early and late effector genes that are expressed by keratinocytes at the wound edge [22].

Other aspects of the repair process, in particular the stopping phase where migratory and proliferative behaviors cease as wound edges confront one another, have not yet been successfully modeled *in vitro*, but our study shows that even in the complexity of *in vivo *healing, many hints as to the genes responsible for these episodes can be gleaned by microarray surveys.

Clearly, the most novel aspect of our study is its capacity to highlight those gene responses that are specifically associated with, or a consequence of, the wound-activated inflammatory response. Those genes expressed at the wound site by virtue of their being expressed by the invading leukocytes provide clues as to the migratory machinery of leukocytes *in vivo*, informing us, for example, which chemokines might be key attractive cues by revealing which of the chemokine receptors are expressed by these cells. Of most therapeutic significance are those genes expressed as a consequence of inflammation by host wound fibroblasts, endothelial and muscle cells. These genes are clearly not absolutely essential for repair, or *PU.1 *skin wound not heal. Rather, they will include genes that contribute to the negative side effects of inflammation at the wound site including retarded re-epithelialization and fibrosis. Dissecting out exactly which genes from the inflammation-associated clusters might sit in such a category will be a major goal of our future studies.

How full a survey of the wound healing process is revealed by our microarray study? The Affymetrix GeneChip we used covered approximately half of the mouse genome and so we cannot claim this to be a saturation screen. Moreover, it has not escaped our attention that several well established wound players that we know to be represented on the chips are apparently absent from any of the wound clusters. This is true for several growth factors, most notably TGFβ1, which we have previously shown to be differentially expressed in wild-type versus *PU.1 *null wounds in RNase protection assays [6], and the same may be true for several other classes of genes expressed at low copy number. A similar observation was made in a recent serial analysis of gene expression (SAGE) study of *Drosophila *genes expressed downstream of JNK signaling, which highlighted many such genes but revealed barely any change in expression of the TGFβ family member *dpp*, for which there is excellent genetic evidence for downstream activation by JNK signaling [47]. For these reasons it is clear that our screen underestimates the numbers of genes associated with each of the repair episodes and is perhaps somewhat biased towards genes expressed at higher copy number.

As we have highlighted throughout this paper, revealing a temporal expression profile that coincides with one of the physiological episodes of the repair process in no way proves function for a wound-expressed gene. Although limited to a small sample of genes, our *in situ *hybridization studies add spatial resolution to this dataset, revealing whether a gene is expressed by the wound epidermis or connective tissue cells, or by inflammatory cells, but given the vast array of genes expressed at the wound site, how can one dissect each of their roles during the repair process? Using recent developments in Cre driver lines, it will be possible to knockout genes specifically in appropriate cell lineages within the mouse, so that the requirement for a particular chemokine receptor in the recruitment of inflammatory cells can be assessed, or the link between expression of a candidate 'fibrosis' gene by wound fibroblasts and subsequent scarring, can be tested. Complementary studies in which mRNAs for candidate inflammation/fibrosis genes are simply knocked down by local delivery to the wound site of antisense oligodeoxynucleotides (AS ODNs) [48] will provide a testbed for whether such approaches may be of therapeutic benefit to improve the repair process. While mammalian models may remain the best guide for potential clinical application, it may be faster and more efficient to turn to simpler, more genetically tractable organisms to trawl through the vast numbers of candidate repair and inflammation genes revealed by microarray studies. Indeed, we and others [49-51] have begun to use *Drosophila *as a testbed to dissect the genetics of some aspects of the repair process and to determine by mutant analysis precisely which genes are required for which repair episodes.

In summary, we present here a portfolio of genes expressed during the *in vivo *wound healing process and categorized according to the physiological episodes that best correlate with their temporal expression profile. Such a classified listing provides good clues to the genetic regulation of all the cell behaviors that contribute to healing, and supplies us with a pool of genes whose modulation may prove to be therapeutically beneficial to the repair process.

## Materials and methods

### Mice and wounding

Generation and PCR genotyping of *PU.1 *mice has been described previously [6]. Tail-tip blood smears were stained with Giesma (Sigma) for rapid identification of null individuals by the absence of neutrophils. Two-day pups received local anesthetic and full-thickness incisional wounds were made to a 1 cm × 0.5 cm area of the back skin. For the microarray study, a criss-cross network of 10 × 8 incisional wounds were made with a scalpel (Figure 1a) such that all cells within the rectangle of skin were adjacent to a cut. For *in situ *hybridization studies, three incisional wounds were made along the long axis of this patch of skin (Figure 1b). Subsequently, *PU.1 *null mice and control sibs were maintained with daily antibiotic injections until sacrifice at appropriate times. For microarray experiments, full-thickness back skin was dissected and immediately transferred, without fixation, into liquid nitrogen for subsequent RNA extraction. Wound harvesting and cryostat tissue sectioning for immunohistochemistry and *in situ *hybridization were performed after perfusion fixation with 4% paraformaldehyde in PBS as described [6] with tissue sections cut at 14 μm.

### RNA extraction and preparation for hybridization

Total RNA was isolated from all skin samples using RNAzol (Biogenesis) according to the manufacturer's instructions with a secondary clean-up stage using an RNeasy kit (Qiagen). RNA was amplified and labeled with biotin as described previously [52].

### Array hybridization and scanning

Double-stranded cDNA was generated from 10 μg total RNA using Superscript Choice kit (Life Technologies) with a T7-poly(T) primer. Approximately 1 μg cDNA was used to generate biotinylated cRNA by *in vitro *transcription using Bioarray High Yield RNA Transcript Labelling kit (Enzo Diagnostics Inc). Fragmented cRNA (10 μg) was hybridized in 100 mM β-mercaptoethanol, 1 M NaCl, 20 mM EDTA, 0.01% Tween20, 0.1 mg/ml herring sperm DNA, 0.5 mg/ml acetylated BSA, 50 pM control oligonucleotide and eukaryotic hybridization controls, to Affymetrix MGU74A arrays at 45°C for 16 h. Arrays were washed using Affymetrix protocols in nonstringent buffer (6x SSPE buffer, 0.01% Tween20, 0.005% antifoam) at 25°C and stringent wash buffer (100 mM MES buffer, 0.1 M NaCl, 0.01%Tween20) at 50°C and stained with streptavidin phycoerythrin (10 μg/ml) including an antibody amplification step. Arrays were scanned using an Affymetrix confocal scanner.

### Analysis of GeneChip data

The data were analyzed using Microarray Analysis Suite version 4.0 (Affymetrix). The data were scaled to a target intensity of 300. Values representing genes not expressed are unreliable and are given an absent call. All genes were subjected to a filter to identify genes with at least one present call across the full eight time points and that had on one or more occasion a greater than twofold change in gene expression levels between time points or between the wild-type and *PU.1 *null. The remaining genes were sorted into nine clusters using Spotfire Array Explorer 3.0 software, of which seven correlated with clear physiological episodes of repair or inflammation and we have loosely named those clusters according to these criteria.

### Resin histology and *c-fms *in situ hybridization

Wound tissues were processed for resin histology with sections cut at 5 μm and stained with Toluidine Blue as previously described [6]. To visualize macrophages we carried out *in situ *hybridization studies using the macrophage-specific *c-fms *probe, using the protocol outlined in [6] and see below.

### *In situ *hybridization

Probe details are available in Additional data file 3. *In situ *hybridization on frozen sections was performed as previously described [53], with probe hybridization carried out in a humidity chamber at 55°C for 16 h. Expression was visualized by BCIP/NBT precipitation (Roche Biochemicals) and sections viewed on a Zeiss Axiphot microscope after mounting in Citifluor (UKC). This spatial expression information gave us a good indication of which were the expressing cell lineages in the wound site, particularly for epidermal keratinocytes (most superficial cell layer), and leukocytes (scattered individual cells in the wound granulation tissue), but without double staining with lineage-specific antibodies we cannot be definite, particularly in distinguishing specific leukocyte lineages from subpopulations of wound fibroblasts and so forth.

## Additional data files

The following additional data are available with the online version of this paper. Additional data file 1, wound gene bioinformatics, is an Excel file containing an annotated database for the 1,001 differentially expressed genes in the nine temporal gene clusters: activation, early effector, late effector, stop, early inflammatory, late inflammatory; inflammation-maintained, and excluded clusters 1 and 2. For each gene this database provides an Affymetrix ID and GenBank description, together with absolute fluorescence levels and absence/presence calls for each time point, and known functional information gained from GenBank and Swiss-Prot databases. Additional data file 2, excluded clusters, contains line graphs displaying the temporal profile of the median expression levels at each time point to give a representation of excluded clusters 1 and 2. Additional data file 3, *in situ *hybridization probe source, is a Word document listing the origins of the various RNA probes used in the *in situ *hybridization studies. Additional data file 4, full array expression data, is an Excel file containing the raw data - absolute fluorescence levels and absence/presence calls - for all genes on the Affymetrix MGU74 arrays at all experimental time points.

## Supplementary Material

Additional data file 1 An Excel file containing an annotated database for the 1,001 differentially expressed genes in the nine temporal gene clusters: activation, early effector, late effector, stop, early inflammatory, late inflammatory; inflammation-maintained, and excluded clusters 1 and 2. For each gene this database provides an Affymetrix ID and GenBank description, together with absolute fluorescence levels and absence/presence calls for each time point, and known functional information gained from GenBank and Swiss-Prot databases Click here for additional data file

Additional data file 2 Line graphs displaying the temporal profile of the median expression levels at each time point to give a representation of excluded clusters 1 and 2 Click here for additional data file

Additional data file 3 A Word document listing the origins of the various RNA probes used in the *in situ * hybridization studies Click here for additional data file

Additional data file 4 An Excel file containing the raw data - absolute fluorescence levels and absence/presence calls - for all genes on the Affymetrix MGU74 arrays at all experimental time points Click here for additional data file
