# Supplementary figures and images for: Wound healing and inflammation genes revealed by array analysis of 'macrophageless' PU.1 null mice
Source: Genome Biol. 2004 Dec 23;6(1):R5. doi: 10.1186/gb-2004-6-1-r5 (PMC549066; doi:10.1186/gb-2004-6-1-r5)

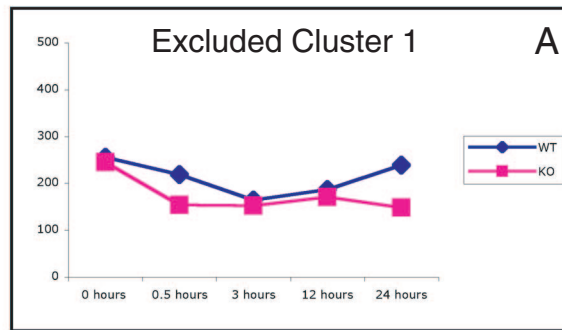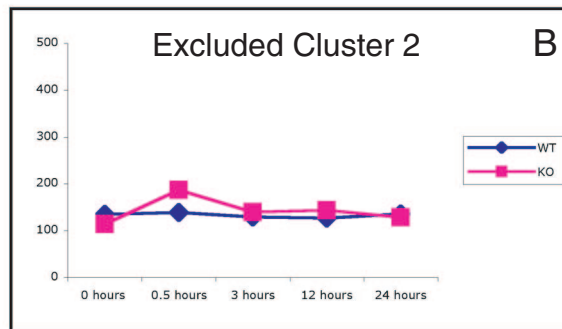

Supplement: Additional data file 2 — Line graphs displaying the temporal profile of the median expression levels at each time point to give a representation of excluded clusters 1 and 2 [file gb-2004-6-1-r5-s2.pdf]
