# Supplementary material for: Wound healing and inflammation genes revealed by array analysis of 'macrophageless' PU.1 null mice
Source: Genome Biol. 2004 Dec 23;6(1):R5. doi: 10.1186/gb-2004-6-1-r5 (PMC549066; doi:10.1186/gb-2004-6-1-r5)
Supplement: Additional data file 3 — A Word document listing the origins of the various RNA probes used in the in situ hybridization studies [file gb-2004-6-1-r5-s3.doc]

*In situ* hybridisation probes source

DNA Source

*Cfms*  [54]

*Krox 24* [55]

*MKP1* PCR cloned

*Fosl1* EST AI225491

*AI853531* EST

*Map4k4* EST AA260451

*Rbp1* EST BI412436

*K6* [19]

*MRP8* PCR cloned

*Notch1* EST BI557031

*L-plastin* EST BF538765

*C3* EST BI554465

*Onzin* EST AA981866

*MRP14* PCR cloned

*Spp1* EST AI325482

*CCr1* EST BI685810

*CXCL10* EST AA154446

*Cathepsin S* EST BC002125

*Repetin* EST AA762506

*Agtr2*  EST BU518263

*Mcpt5*  EST AA060135

*CCL2*  EST BE686563

*CCL7*  EST AA711435
